# Total glucosides of paeony inhibits lipopolysaccharide-induced proliferation, migration and invasion in androgen insensitive prostate cancer cells

**DOI:** 10.1371/journal.pone.0182584

**Published:** 2017-08-04

**Authors:** Zhi-Hui Zhang, Dong-Dong Xie, Shen Xu, Mi-Zhen Xia, Zhi-Qiang Zhang, Hao Geng, Lei Chen, Da-Ming Wang, Wei Wei, De-Xin Yu, De-Xiang Xu

**Affiliations:** 1 Department of Toxicology, School of Public Health, Anhui Medical University, Hefei, Anhui Province, China; 2 Department of Urology, Second Affiliated Hospital, Anhui Medical University, Hefei, Anhui Province, China; 3 School of Biological Sciences, Anhui Medical University, Hefei, Anhui Province, China; 4 Institute of Clinical Pharmacology, Anhui Medical University, Key Laboratory of Anti-inflammatory and Immunopharmacology of Education Ministry, Hefei, Anhui Province, China; Duke University School of Medicine, UNITED STATES

## Abstract

Previous studies demonstrated that inflammatory microenvironment promoted prostate cancer progression. This study investigated whether total glucosides of paeony (TGP), the active constituents extracted from the root of *Paeonia Lactiflora Pall*, suppressed lipopolysaccharide (LPS)-stimulated proliferation, migration and invasion in androgen insensitive prostate cancer cells. PC-3 cells were incubated with LPS (2.0 μg/mL) in the absence or presence of TGP (312.5 μg /mL). As expected, cells at S phase and nuclear CyclinD1, the markers of cell proliferation, were increased in LPS-stimulated PC-3 cells. Migration activity, as determined by wound-healing assay and transwell migration assay, and invasion activity, as determined by transwell invasion assay, were elevated in LPS-stimulated PC-3 cells. Interestingly, TGP suppressed LPS-stimulated PC-3 cells proliferation. Moreover, TGP inhibited LPS-stimulated migration and invasion of PC-3 cells. Additional experiment showed that TGP inhibited activation of nuclear factor kappa B (NF-κB) and mitogen-activated protein kinase (MAPK)/p38 in LPS-stimulated PC-3 cells. Correspondingly, TGP attenuated upregulation of interleukin (IL)-6 and IL-8 in LPS-stimulated PC-3 cells. In addition, TGP inhibited nuclear translocation of signal transducer and activator of transcription 3 (STAT3) in LPS-stimulated PC-3 cells. These results suggest that TGP inhibits inflammation-associated STAT3 activation and proliferation, migration and invasion in androgen insensitive prostate cancer cells.

## Introduction

In Western countries, prostate cancer is one of the most common malignant tumors in men [1]. The prevalence of prostate cancer differs from one country to another due to coverage of prostate-specific antigen screening [2]. In China, the rate of prostate cancer is rapidly increasing and especially in patients with obesity or diabetes [3,4]. As androgen receptor plays an important role in the pathogenesis of prostate cancer, androgen-deprivation therapy remains the principal method for treatment of patients with prostate cancer [5]. Unfortunately, the majority of patients with advanced-stage prostate cancer will ultimately progress from castration-sensitive to castration-resistant prostate cancer [6]. Recently, several studies demonstrate that inflammatory microenvironment promotes prostate cancer development and progression [7–9]. Nuclear factor kappa B (NF-κB) and nuclear translocation of signal transducer and activator of transcription (STAT)3 play important roles in the pathogenesis of inflammation-associated progression of prostate cancer.

The total glucosides of paeony (TGP), the active constituents extracted from the root of *Paeonia Lactiflora Pall*, have been used as a traditional Chinese medicine for the treatment of rheumatoid arthritis [10,11]. Recently, several studies found that TGP could effectively alleviate the progression of diabetic nephropathy [12,13]. Accumulating evidence demonstrates that TGP has potential anti-inflammatory activities [14,15]. An early study showed that TGP suppressed inflammatory cytokines and mediators in macrophage-like synoviocytes from rats with adjuvant arthritis [16]. Nevertheless, whether TGP curbs inflammation-associated prostate cancer progression remains to be determined.

The primary objective of the present study was to evaluate whether TGP suppresses LPS-evoked proliferation, migration and invasion in PC-3 cell, an androgen insensitive prostate cancer cell line. Moreover, we were to investigate whether TGP inhibits LPS-induced activation of NF-κB signaling. Finally, we were to investigate the inhibitive effects of TGP on LPS-induced IL-6 and IL-8 and subsequent activation of STAT3 signaling in PC-3 cells.

## Materials and methods

### Drugs and reagents

Total glucosides of paeony (TGP) was from Institute of Clinical Pharmacology, Anhui Medical University (Anhui, China) [10]. Lipopolysaccharide (*Escherichia coli* LPS, serotype 0127:B8) was purchased from Sigma Chemical Co. (St. Louis, MO). Antibodies against CyclinD1, phosphor-p38 (p-p38), p38, NF-κB p50 and pSTAT3 were from Cell Signaling Technology (Beverley, MA). Antibodies against STAT3, p-IκB, I-κB, NF-κB p65, and Lamin A/C were from Santa Cruz Biotechnologies (Santa Cruz, CA). TRI reagent was from Molecular Research Center, Inc (Cincinnati, Ohio). RNase-free DNase was from Promega Corporation (Madison, WI). Chemiluminescence detection kit was from Pierce Biotechnology (Rockford, IL). All other reagents were purchased from Sigma Chemical Co. (St. Louis, MO) if not otherwise stated.

### Cell culture and treatments

The androgen insenstitive prostate cancer cell line PC-3 cell was obtained from the Cell Bank of the Chinese Academy of Sciences (Shanghai, China). Cells were grown in T25 cell culture flasks (Corning) in F-12 HAM’S Medium (HyClone) supplemented with 100 U/mL of penicillin, 100 μg/mL streptomycin and 10% FBS (Gibco) at 5% CO_2_, 37°C. The cells were incubated for at least 24 hr to allow them to adhere to the plates. About 80% confluent, the medium was replaced with serum-free medium. After a 4 hr incubation, the cells were incubated with LPS (2.0 μg/mL) for different times in the absence or presence of TGP (312.5 μg/mL). The cells were washed with chilled PBS for three times and then harvested for real-time RT-PCR and immunoblots.

### Cell cycle analysis

The effect of TGP on cell cycle was performed as described previously by MUSE^®^ Cell Analyzer (Merck Millipore, Germany) [17]. Briefly, 3.0 × 10^5^ cells were cultured in 6-well plates until 80% confluent. The medium was replaced with serum-free medium (containing corresponding drugs). After 6 hr incubation, cells were harvested and counted. Around 1 × 10^6^ cells were transferred to a 2 mL tube. The cells were centrifuged at 300 × g for 5 min and washed twice with PBS. The washed cells were fixed with ice cold 70% ethanol. For fixation, cells were incubated overnight at −20°C. 200 μL of fixed cells were centrifuged at 300 × g for 5 min and washed twice with PBS. The cells were mixed with 200 μL of Muse^®^ Cell Cycle Assay Kit (Merck Millipore, Germany) and incubated for 30 min at room temperature in dark. Cell cycle was analyzed using Muse cell analyzer.

### Cell viability and apoptosis analysis

The effects of TGP on cell viability and apoptosis were evaluated as described previously by MUSE^®^ Cell Analyzer [18]. Briefly, 3.0 × 10^5^ cells were cultured in a 6-well plates until 80% confluent. The medium was replaced with serum-free medium (containing the corresponding drugs). After 6 hr incubation, cells were harvested and counted. For cell viability, 20 μL (3.0 × 10^5^) of cell suspension and 380 μL of Muse™ Count & Viability Reagent (Merck Millipore, Germany) were mixed and incubated for 5 min at room temperature in dark. For cell apoptosis, 100 μL (3.0 × 10^5^) of cell suspension and an equal volume of Muse™ Annexin V & Dead Cell Reagent (Merck Millipore, Germany) were mixed and incubated for 20 min at room temperature in dark. Cell viability and apoptosis were analyzed using Muse cell analyzer.

### Wound healing migration assay

Wound healing assay was performed as described previously with minor modifications [19]. Briefly, cells (5.0 × 10^5^ cells per well) were cultured in a 6-well plates until 80% confluent. The medium was replaced with serum-free medium (containing corresponding drugs). After 12 hr incubation, the medium was collected. The confluent monolayer cells were carefully scratched using a 200 μL tip and washed twice with PBS. Previous mediums were added to corresponding wells. The cells were photographed at low magnification for time intervals of 0, 12 and 24 hr. The wounded area was calculated according to the formula: (mean wounded breadth—mean remained breadth)/mean wounded breadth × 100 (%). The experiment was carried out three times independently.

### Cell migration and invasion assays

Migration and invasion activities of PC-3 cells were evaluated using 8-μm Transwell filters (Costar Corning, Schiphol-Rijk, Netherlands) with minor modifications as described previously [20]. For migration assay, 4 × 10^4^ cells in 0.2 mL complete medium were seeded in upper compartment. The plates were incubated for 24 hr at 37°C, 5% CO_2_. After 24 hr incubation, the complete medium in upper chamber was replaced with serum-free medium (supplement with 0.5% BSA and drugs). The lower compartment was filled with 0.6 mL basal medium containing 10% FBS as chemoattractant and then incubated for 24 hr. For invasion assay, 4 × 10^4^ cells in 0.2 mL complete medium were seeded in upper compartment precoated with 50 μL Matrigel solution (100 μg/mL, BD Biosciences, San Jose, CA). After 24 hr incubation, the complete medium in upper chamber was replaced with serum-free medium (supplement with 0.5% BSA and drugs). The lower compartment was filled with 0.6 mL basal medium containing 10% FBS as chemoattractant and then incubated for 48 hr. The non-migratory cells on upper side of chamber and medium of lower chamber were removed. The membranes were fixed with methanol for 20 min and stained with 0.1% crystal violet solution for 20 min. The numbers of cells that had migrated to the bottom of the filter were evaluated by detecting the absorbance of decolorization solution of 30% acetic acid at 570 nm. These experiments were performed three times independently.

### Isolation of total RNA and real-time RT-PCR

Total RNA was extracted from PC-3 cells using TRI reagent according to the manufacturer’s instructions. Real-time RT-PCR was performed as described in detail previously [21]. Briefly, total RNA (1.0 μg) was reverse-transcribed with AMV (Promega). Real-time RT-PCR was performed with a LightCycler® 480 SYBR Green I kit (Roche Diagnostics GmbH, Mannheim, Germany) using gene-specific primers as listed in Table 1. The amplification reactions were carried out on a LightCycler® 480 Instrument (Roche Diagnostics GmbH) with an initial hold step (95°C for 5 minutes) and 50 cycles of a three-step PCR (95°C for 15 seconds, 60°C for 15 seconds, 72°C for 30 seconds). The comparative CT-method was used to determine the amount of target, normalized to an endogenous reference (18S) and relative to a calibrator using the LightCycler 480 software (Roche, version 1.5.0).

**Table 1:** Oligonucleotide sequence of primers for real-time RT-PCR.

Genes	Forward (5’-3’)	Reverse (5’-3’)
*18S*	CGGCTACCACATCCAAGGAA	GCTGGAATTACCGCGGCT
*MMP-3*	CGGTTCCGCCTGTCTCAAG	CGCCAAAAGTGCCTGTCTT
*MMP-9*	TGTACCGCTATGGTTACACTCG	GGCAGGGACAGTTGCTTCT
*u-PA*	GGGAATGGTCACTTTTACCGAG	GGGCATGGTACGTTTGCTG
*IL-6*	AGACAGCCACTCACCTCTTCAG	TTCTGCCAGTGCCTCTTTGCTG
*IL-8*	ACCACCGGAAGGAACCATCT	AGCACTCCTTGGCAAAACTG

### Enzyme-linked immunosorbent assay

Commercial ELISA kits (4A Biotech Co. Ltd. Beijing, China) were used to determine IL-6 and IL-8 levels according to manufacturer's protocol.

### Cytoplasmic protein and nuclear protein extraction

Cytoplasmic protein and nuclear protein were extracted from cells using a method by Arash Nabbi and Karl Riabowol [22,23]. Briefly, PC-3 cells grown in 10-cm dishes were washed in ice-cold PBS (137 mM NaCl, 2.7 mM KCl, 10 mM Na_2_HPO_4_, 1.8 mM KH_2_PO_4_, pH 7.4), scraped from culture dishes on ice using a plastic cell scraper and transferred the cells to a 1.5 mL micro-centrifuge tubes. Supernatants were removed after centrifugation (“pop-spin” for 10 sec at 10,000 × rpm) and cell pellets resuspended in 400 μL of ice-cold PBS containing 0.1% NP-40 supplemented with a cocktail of protease inhibitors (Roche). Triturated 10 times using a p1000 micropipette tip and centrifuged for 20 sec at 10,000 × rpm. The supernatants were removed as the “cytoplasmic protein”. The remaining pellet was washed in 1 mL of PBS containing 0.1% NP40 and centrifuged as above for 20 sec and the supernatant was discarded. Resuspend the nuclear pellet in 150 μL lysis buffer (50 mM Tris-HCl, pH 7.4, 150 mM NaCl, 1 mM EDTA, 1% Triton X-100, 1% sodium deoxycholate, 0.1% sodium dodecylsylphate, 1 mM phenylmethylsulfonyl fluoride) in the presence of protease inhibitor cocktail, incubated on ice for 30 min, and centrifuged for 10 min at 14,000 × g. The supernatants were removed as the “nuclear protein”. Protein concentrations were determined with BCA protein assay (Pierce, Rockford, IL, USA) according to instruction.

### Western blotting

Western blotting was analyzed as described in detail previously [21]. Briefly, same amount of protein (10~20 μg) was separated electrophoretically by SDS-PAGE and transferred to a PVDF membrane. The membranes were incubated for 2 hr with following antibodies: CyclinD1 (1:2000), pRb (1:1000) and PCNA (1:1000), pI-κB (1:2000), I-κB (1:2000), pSTAT3 (1:1000), STAT3 (1:1000), p-p38 (1:1000), p38 (1:1000), NF-κB p50 (1:2000), and NF-κB p65 (1:1000). For nuclear protein, Lamin A/C (1:2000) was used as a loading control. After washed in DPBS containing 0.05% Tween-20 four times for 10 min each, the membranes were incubated with goat anti–rabbit IgG or goat anti–mouse antibody for 2 hr. The membranes were then washed for four times in DPBS containing 0.05% Tween-20 for 10 min each, followed by signal development using an ECL detection kit.

### Statistical analysis

All data were expressed as means ± SEM. SPSS 13.0 statistical software was used for statistical analysis. All statistical tests were two-sided using an alpha level of 0.05. ANOVA and the Student-Newmann-Keuls post hoc test were used to determine differences among different groups.

## Results

### TGP inhibits proliferation of LPS-stimulated PC-3 cells

**Fig 1:**
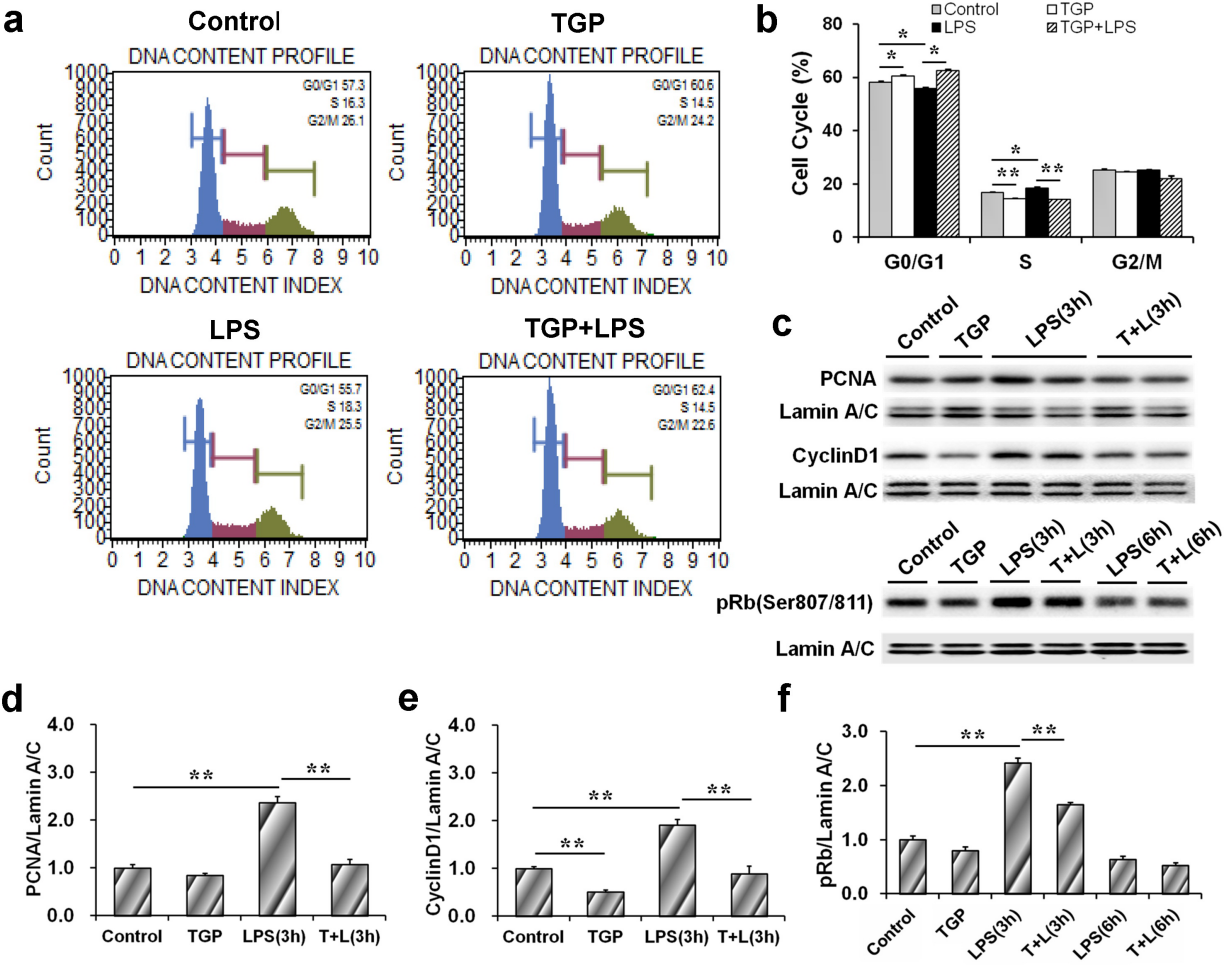
TGP inhibits cell proliferation of LPS-stimulated PC-3 cells. PC-3 cells were incubated with LPS (2.0 μg/mL) in absence or presence of TGP (312.5 μg/mL). Cells were collected at 6 hr after LPS. (a) Cell cycle was measured using Muse™ Cell Cycle Kit. (b) Cell quantification of each cell cycle phase. (c-f) Cells were collected at 3 hr and 6 hr after LPS. (c) A representative gel for PCNA (upper panel), CyclinD1 (middle panel) and pRb (lower panel) was shown. (d) PCNA/Lamin A/C, (e) CyclinD1/Lamin A/C and (f) pRb/Lamin A/C. All experiments were repeated for three times. Data were expressed as means ± S.E.M. (N = 3), *P<0.05, **P<0.01.

The effects of TGP on LPS-stimulated proliferation of PC-3 cells were analyzed. As expected, the percentage of cells at G0/G1 phase was significantly reduced in LPS-stimulated cells. In contrast, the percentage of cells at S phase was elevated in LPS-stimulated cells (Fig 1A and 1B). As shown in Fig 1C, nuclear CyclinD1 level was elevated in LPS-stimulated cells. Moreover, nuclear PCNA and pRb levels were elevated as early as 3 hr after LPS treatment (Fig 1D and 1F). Interestingly, the percentage of cells at S phase was reduced when LPS-stimulated PC-3 cells were incubated with TGP (Fig 1A and 1B). In addition, LPS-induced nuclear translocation of CyclinD1, pRb and PCNA was suppressed by TGP (Fig 1C and 1F).

### TGP does not affect PC-3 cell apoptosis

**Fig 2:**
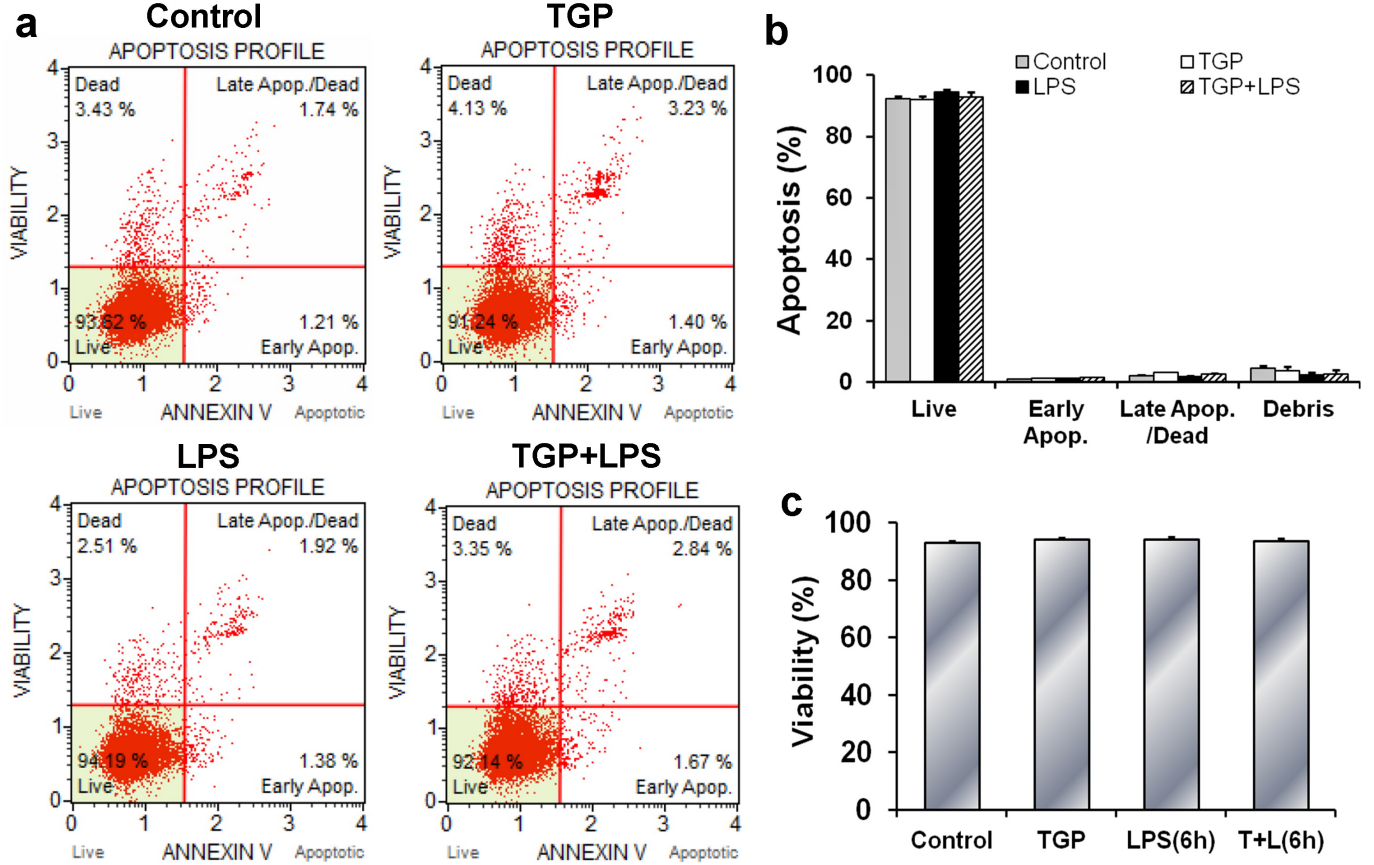
Effects of TGP on cell apoptosis and viability in LPS-stimulated PC-3 cells. PC-3 cells were incubated with LPS (2.0 μg/mL) in absence or presence of TGP (312.5 μg/mL). Cells were collected 6 hr after LPS. (a) Cell apoptosis was measured using Muse™ Annexin V & Dead Cell Kit. (b) Quantification of cell apoptosis. (c) Cell viability was evaluated using Muse™ Count & Viability Kit. All experiments were repeated for three times. Data were expressed as means ± S.E.M. (N = 3), *P<0.05, **P<0.01.

The effects of TGP on apoptosis of PC-3 cells were analyzed. As shown in Fig 2, LPS had little effect on PC-3 cell apoptosis. In addition, TGP does not affect apoptosis and viability of LPS-stimulated PC-3 cells (Fig 2).

### TGP inhibits migration of LPS-stimulated PC-3 cells

**Fig 3:**
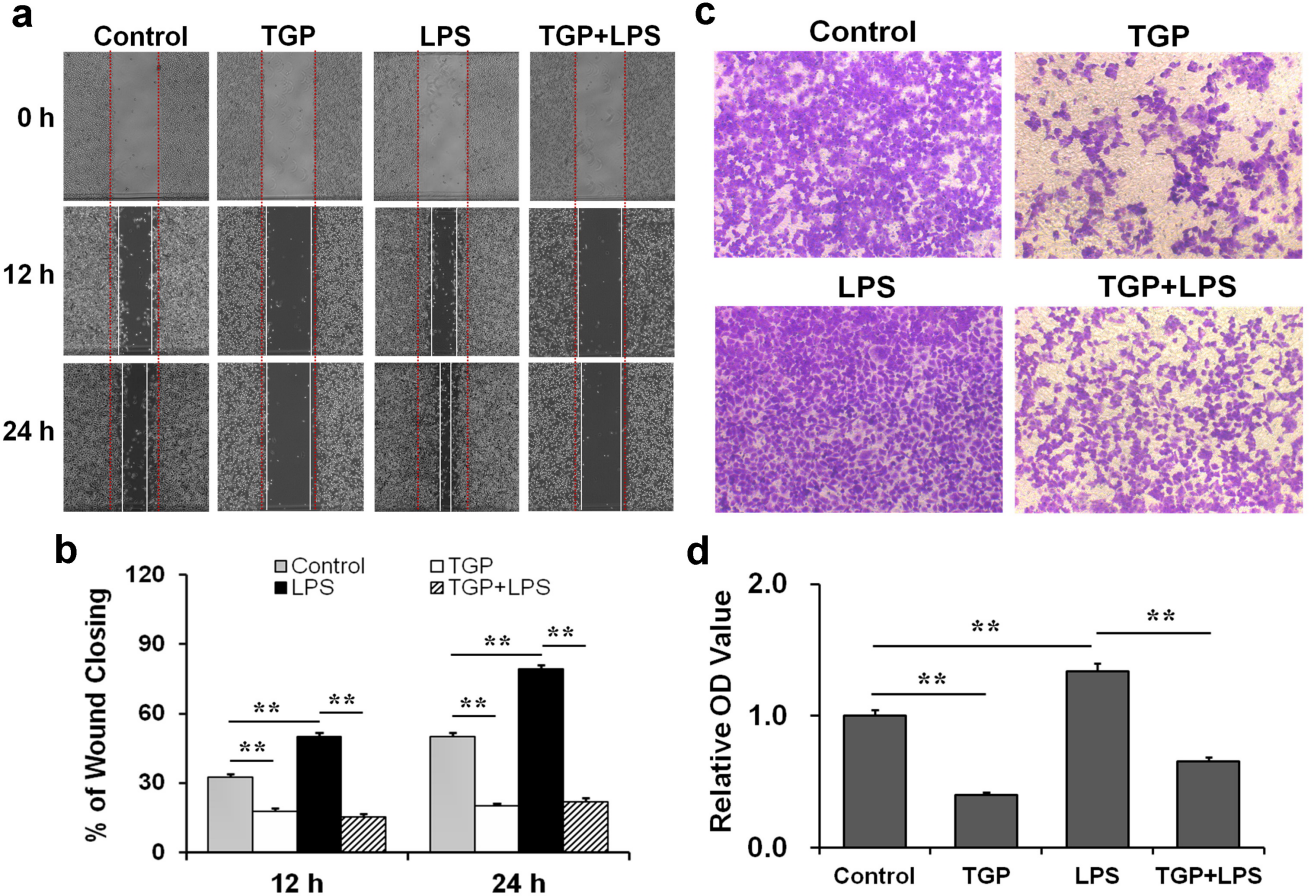
TGP inhibits migration of LPS-stimulated PC-3 cells. PC-3 cells were incubated with LPS (2.0 μg/mL) in absence or presence of TGP (312.5 μg/mL). (a) The migration of PC-3 cells was measured using wound-healing assay. After 0 hr, 12 hr and 24 hr migration, the scratches were photographed. (b) The wounded areas after 12 hr and 24 hr incubations were calculated as described in the Materials and Methods. (c) The migration of PC-3 cells was measured using transwell migration assay. (d) The numbers of migrated cells were indirectly evaluated as described in the Materials and Methods. All experiments were repeated for three times. Data were expressed as means ± S.E.M. (N = 3), **P<0.01.

The effects of TGP on LPS-stimulated migration of PC-3 cells were analyzed. Wound-healing assay showed that the percentage of wound closing was significantly increased in LPS-stimulated PC-3 cells (3A and 3B). Correspondingly, cell migration, as determined by the transwell migration assay, was elevated in LPS-stimulated PC-3 cells (Fig 3C and 3D). Interestingly, TGP alone inhibited migration of PC-3 cells (Fig 3A–3D). Moreover, TGP also blocked migration of LPS-stimulated PC-3 cells (Fig 3A–3D).

### TGP inhibits invasion of LPS-stimulated PC-3 cells

**Fig 4:**
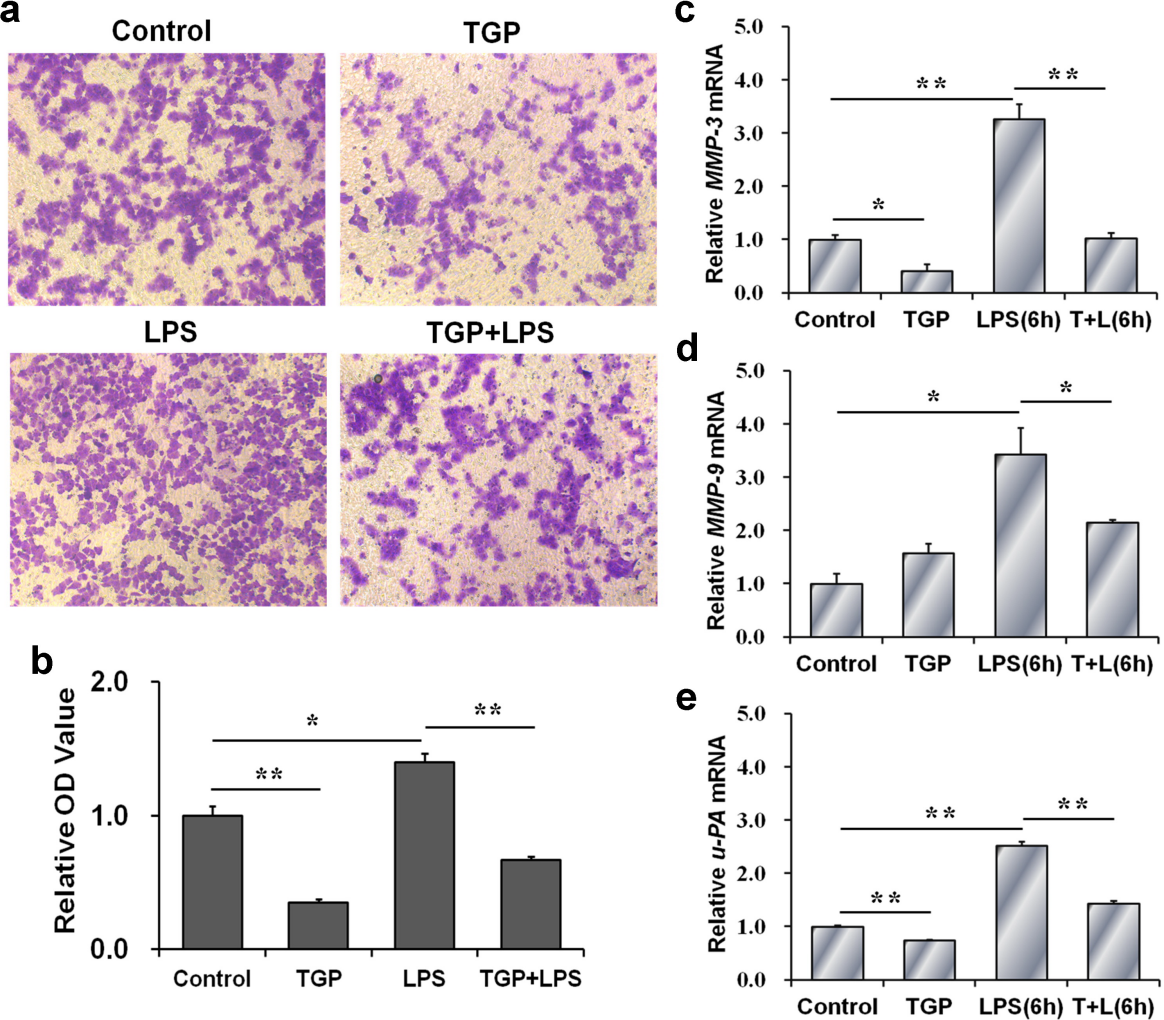
TGP inhibits invasion of LPS-stimulated PC-3 cells. PC-3 cells were incubated with LPS (2.0 μg/mL) in absence or presence of TGP (312.5 μg/mL). (a) The invasion of PC-3 cells was measured using transwell invasion assay. (b) The numbers of migrated cells were indirectly evaluated as described in the Materials and Methods. (c-e) Cells were collected at 6 hr after LPS. MMP-3 (c), MMP-9 (d) and u-PA (e) mRNAs were determined using RT-PCR. All experiments were repeated for three times. Data were expressed as means ± S.E.M. (N = 3), *P<0.05, **P<0.01.

The effects of TGP on invasion of LPS-stimulated PC-3 cells were analyzed. As expected, cell invasion, as determined by transwell invasion assay, was significantly increased in LPS-stimulated PC-3 cells (Fig 4A and 4B). Moreover, mRNA levels of MMP-3 and MMP-9 were elevated in LPS-stimulated PC-3 cells (Fig 4C and 4D). In addition, u-PA mRNA was up-regulated in LPS-stimulated PC-3 cells (Fig 4E). Interestingly, TGP alone obviously repressed cell invasion of PC-3 cells. Moreover, TGP also inhibited invasion in LPS-stimulated PC-3 cells (Fig 4A and 4B). In addition, TGP inhibited LPS-induced up-regulation of MMP-3, MMP-9 and u-PA in PC-3 cells (Fig 4C–4E).

### TGP inhibits LPS-induced up-regulation of IL-6 and IL-8 in PC-3 cells

**Fig 5:**
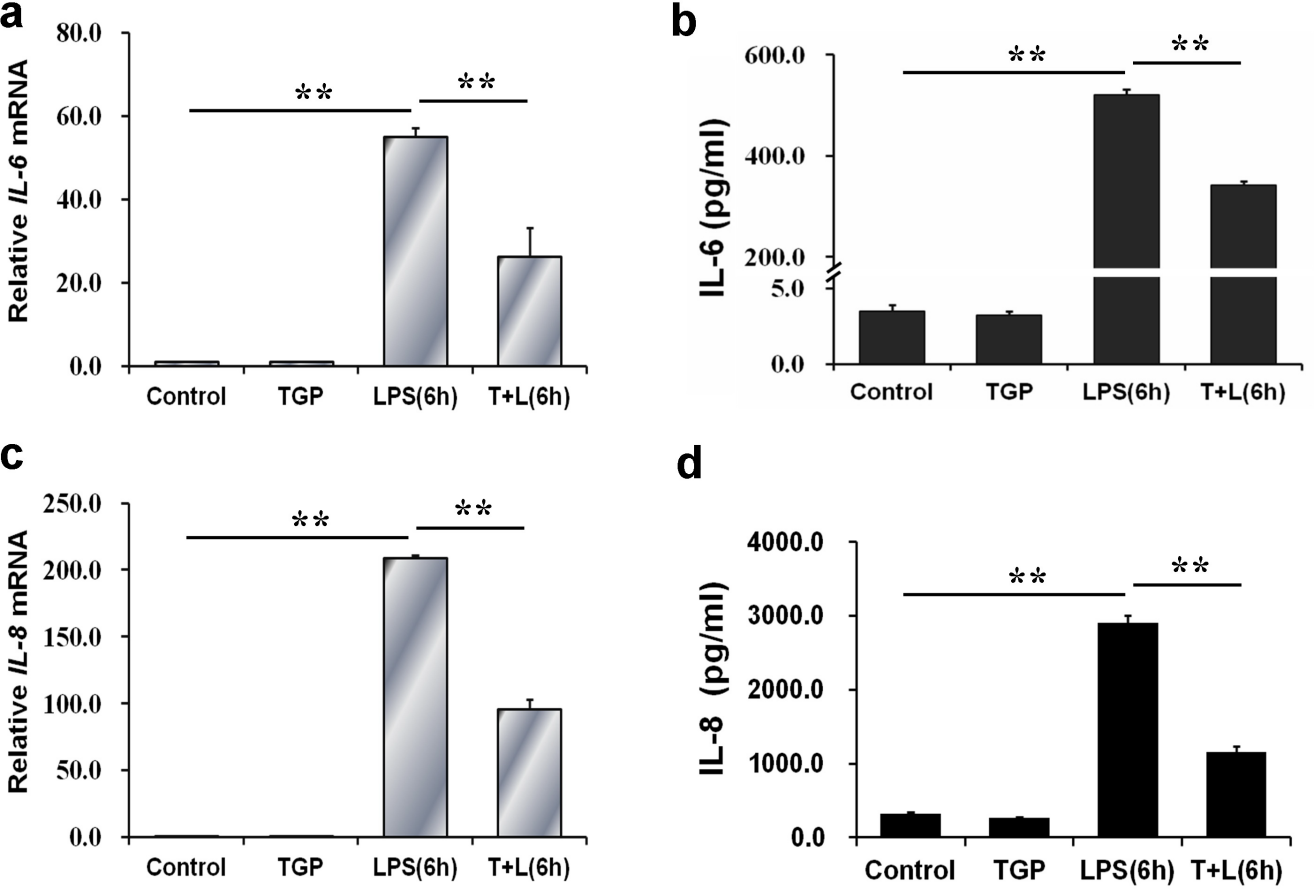
TGP inhibits LPS-evoked IL-6 and IL-8 in PC-3 cells. PC-3 cells were incubated with LPS (2.0 μg/mL) in absence or presence of TGP (312.5 μg/mL). Cells and cultural supernatants were collected at 6 hr after LPS. (a) IL-6 and (c) IL-8 mRNAs were determined using RT-PCR. (b) IL-6 and (d) IL-8 in culture medium were measured using ELISA. Data were expressed as means ± S.E.M. (N = 6), **P<0.01.

The effects of TGP on LPS-induced IL-6 and IL-8 were analyzed. As expected, IL-6 mRNA was up-regulated by about 60 folds in LPS-stimulated PC-3 cells (Fig 5A). In addition, IL-8 mRNA was up-regulated by 200 folds in LPS-stimulated PC-3 cells (Fig 5C). Correspondingly, the levels of IL-6 and IL-8 in culture medium were significantly elevated in LPS-stimulated PC-3 cells (Fig 5B and 5D). Although TGP alone had no effect on IL-6 and IL-8, it significantly attenuated LPS-induced up-regulation of IL-6 and IL-8 in PC-3 cells (Fig 5A–5D).

### TGP inhibits LPS-induced activation of NF-κB and p38/MAPK in PC-3 cells

**Fig 6:**
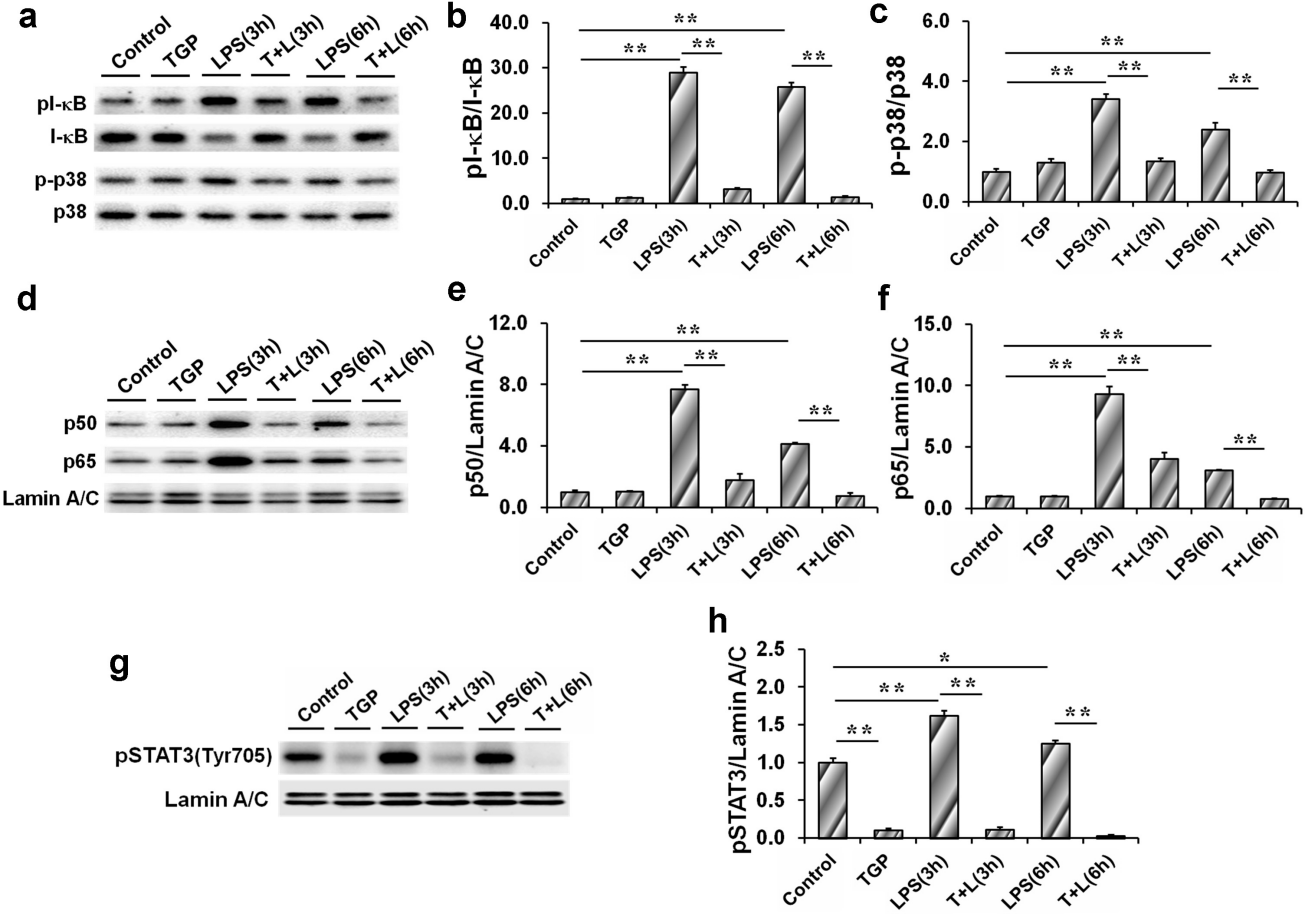
TGP inhibits LPS-evoked activation of NF-κB, p38/MAPK and STAT3 in PC-3 cells. PC-3 cells were incubated with LPS (2.0 μg/mL) in absence or presence of TGP (312.5 μg/mL). Cells were collected 3 hr and 6 hr after LPS. (a) Cytoplasmic p-p38 and pI-κB were determined using immunoblot. (b) pI-κB/I-κB. (c) p-p38/p38. (d) Nuclear NF-κB p50 and p65 subunits were determined using immunoblot. (e) p50/Lamin A/C. (f) p65/Lamin A/C. (g) Nuclear pSTAT3 were determined using immunoblot. (h) pSTAT3/Lamin A/C. All data were expressed as means ± S.E.M. from three experiments (N = 3), *P<0.05, **P<0.01.

The effects of TGP on LPS-induced activation of NF-κB and p38/MAPK were analyzed. As expected, the levels of cytoplasmic p-p38 and pI-κB in PC-3 cells were obviously elevated at 3 hr after LPS treatment and remained increased at 6 hr after LPS treatment (Fig 6A–6C). In addition, the levels of nuclear NF-κB p50 and p65 subunits were elevated in LPS-stimulated PC-3 cells (Fig 6D–6F). Interestingly, TGP significantly inhibited LPS-induced p38/MAPK and I-κB phosphorylation in PC-3 cells (Fig 6A–6C). In addition, TGP blocked nuclear translocation of NF-κB p65 and p50 subunits in LPS-stimulated PC-3 cells (Fig 6D–6F).

### TGP inhibits nuclear translocation of STAT3 in LPS-stimulated PC-3 cells

The effects of TGP on nuclear translocation of STAT3 in LPS-stimulated PC-3 cells were analyzed. As expected, the level of nuclear pSTAT3 was significantly elevated 3 hr after LPS treatment and remained increased 6 hr after LPS treatment (Fig 6G and 6H). Interestingly, TGP alone significantly inhibited nuclear translocation of pSTAT3 in PC-3 cells (Fig 6G and 6H). Moreover, TGP inhibited LPS-induced nuclear translocation of pSTAT3 in PC-3 cells (Fig 6G and 6H).

## Discussion

Previous studies found that TGP, the active constituents extracted from a traditional Chinese herb, had an anti-inflammatory activity [14,15]. The present study investigated whether TGP inhibits inflammation-induced proliferation, migration and invasion in PC-3 cells. As expected, the percentage of cells at S phase was elevated in LPS-stimulated PC-3 cells. Correspondingly, the levels of nuclear CyclinD1 and PCNA, two markers of cell proliferation, were increased in LPS-stimulated PC-3 cells. The migration and invasion activity were elevated in LPS-stimulated PC-3 cells. Interestingly, LPS-stimulated PC cell proliferation was suppressed by TGP. In addition, LPS-stimulated migration and invasion of PC-3 cells were blocked when PC-3 cells were simultaneously incubated with TGP. These results suggest that TGP inhibits not only LPS-stimulated proliferation but also migration and invasion of PC-3 cells.

IL-6 has been associated with prostate cancer progression [24–26]. Indeed, serum IL-6 levels in patients with metastatic prostate cancer were higher than those in patients with localized prostate cancer [27]. In addition, circulating IL-6 level was associated with progression and death in patients with prostate cancer [28]. Several reports from in vitro studies showed that IL-6 up-regulated prostate-specific antigen mRNA and promoted androgen-independent growth in human prostate cancer cells [29,30]. Anti-IL-6 monoclonal antibody suppressed the progression from androgen-dependent prostate cancer to an androgen-independent prostate cancer in orchiectomized mice [31]. In the present study, we analyzed IL-6 expression in LPS-stimulated PC-3 cells. Surprisingly, IL-6 mRNA in PC-3 cells was up-regulated by about 60 folds 6 hr after LPS incubation. Correspondingly, IL-6 level was increased by more than 100 folds in culture medium from LPS-stimulated PC-3 cells. Of interest, LPS-induced up-regulation of IL-6 was attenuated when PC-3 cells were simultaneously incubated with TGP. These results suggest that TGP inhibits LPS-stimulated proliferation, migration and invasion, at least partially, through suppressing IL-6 produced by PC-3 cells.

Several studies showed that IL-8 was highly expressed in prostate cancer [32]. In addition, the levels of IL-8 expression in prostate cancer were higher in the recurred patients than those in the non-recurred patients [33]. Indeed, in patients with metastatic prostate cancer starting androgen-deprivation therapy, a higher level of serum IL-8 was associated with a poorer overall survival [34]. Several early studies showed that IL-8 promoted growth and metastasis of androgen-independent prostate cancer [35,36]. According to a recent report, autocrine IL-8 sustained growth and survival of PTEN-deficient prostate cells [37]. Moreover, tumor-derived IL-8 amplified stroma-derived CCL2-stimulated proliferation and promoted CXCL12-mediated invasion of PTEN-deficient prostate cancer cells [38]. In the present study, our results showed that IL-8 mRNA was up-regulated by more than 200 folds at 6 hr after LPS incubation. Of interest, LPS-induced up-regulation of IL-8 mRNA in PC-3 cells was suppressed by TGP. Moreover, LPS-stimulated release of IL-8 was significantly attenuated in culture medium when PC-3 cells were incubated with TGP. The present study suggests that TGP inhibits LPS-induced prostate cancer progression partially through inhibiting autocrine IL-8.

Accumulating evidence has demonstrated that NF-κB signaling provides a mechanistic link between inflammation and cancer [39]. Indeed, NF-κB signaling was activated in castration-resistant prostate cancer patients [40]. An early study found that NF-κB activation promoted progression of prostate cancer to androgen-independent growth [41]. Several recent studies demonstrated that NF-κB activation contributed to the metastasis of prostate cancer [42,43]. By contrast, blockade of NF-κB signaling in human prostate cancer cells was associated with suppression of angiogenesis, invasion, and metastasis [44]. The present study investigated the effects of TGP on LPS-stimulated NF-κB activation in PC-3 cells. Consistent with its inhibition of inflammatory cytokines, TGP suppressed LPS-induced I-κB phosphorylation in PC-3 cells. In addition, TGP blocked nuclear translocation of NF-κB p65 and p50 subunits in LPS-stimulated PC-3 cells. These results suggest that TGP inhibits inflammation-associated progression of prostate cancer through suppressing NF-κB signaling.

In addition to the classical IL-6/STAT3 pathway, IL-8 also activates JAK2-dependent STAT3 signaling [45,46]. Considerable evidence suggests that activated STAT3 pathway is implicated in the progression of prostate cancer [47]. Indeed, persistent STAT3 activation changed cellular phenotype of benign prostate cells to a malignant one in prostate cancer [48]. In addition, IL-6-driven metastasis of prostate cancer was predominantly mediated by STAT3 signaling [49]. Several early reports demonstrated that down-regulation of STAT3 suppressed the growth of prostate cancer cells [50–52]. Recently, several reports found that the STAT3 inhibitors could effectively reduce tumor growth and metastasis in mouse models of prostate cancer [53,54]. The present study investigated the effects of TGP on LPS-activated STAT3 signaling in PC-3 cells. Surprisingly, TGP alone suppressed activation of STAT3 signaling in PC-3 cells. Moreover, TGP completely blocked LPS-evoked nuclear translocation of STAT3 in PC-3 cells. These results suggest that TGP inhibits LPS-induced progression of prostate cancer partially through suppressing STAT3 pathway.

Several studies demonstrate that inflammation plays an important role in the progression of androgen insensitive prostate cancer cells. The primary objective of the present study was to investigate whether TGP has inhibitory effects on inflammation-evoked proliferation, migration and invasion in PC-3 cells. Although the antagonistic effects of TGP on inflammation-evoked proliferation, migration and invasion in PC-3 cells were discussed for the first time, there are several limitations in the present study. First, the concentration of TGP used referred to other [11] in the present study. In that study, the anti-inflammatory activity of TGP was concentration- dependent and was the strongest at a concentration of 312.5 μg/ml. The present study did not explore the concentration-effect relationship of TGP on inflammation-evoked proliferation, migration and invasion in PC-3 cells. Second, the present study did not explore the effects of TGP on inflammation-evoked proliferation, migration and invasion of other prostate cancer cell lines. Third, the present study did not investigate the effects of TGP on proliferation and metastasis of prostate cancer cells *in vivo*. TGP is a mixture consisting of paeoniflorin, hydroxypaeoniflorin, paeonin, benzoylpaeoniflorin, albiflorin, etc. Complex component and low solubility in water are not conducive to elucidating their exact molecular mechanisms in vivo experiments. Paeoniflorin was one of main effective component of TGP. Thus, the effects of phenformin on inflammation-induced androgen-insensitive prostate cancer progression *in vitro* and *in vivo* will be carried out in other studies. In addition, the clinical relevance of these findings needs to be determined in the future research.

In summary, the present study investigated the effects of TGP on LPS-induced proliferation, migration and invasion in prostate cancer cells. Our results showed that TGP inhibited LPS-induced activation of NF-κB in PC-3 cells. Moreover, TGP inhibited LPS-induced IL-6 and IL-8 and subsequent activation of STAT3 signaling in PC-3 cells. Importantly, TGP inhibited LPS-evoked proliferation, migration and invasion in LPS-stimulated PC-3 cells.

## References

[1] Torre LA, Bray F, Siegel RL, Ferlay J, Lortet-Tieulent J, Jemal A (2015) Global cancer statistics, 2012. CA Cancer J Clin 65: 87–108. doi: 10.3322/caac.21262 25651787 10.3322/caac.21262

[2] Hayes JH, Barry MJ (2014) Screening for prostate cancer with the prostate-specific antigen test: a review of current evidence. JAMA 311: 1143–1149. doi: 10.1001/jama.2014.2085 24643604 10.1001/jama.2014.2085

[3] Hu MB, Bai PD, Wu YS, Zhang LM, Xu H, Na R, et al (2015) Higher body mass index increases the risk for biopsy-mediated detection of prostate cancer in Chinese men. PLoS One 10: e0124668 doi: 10.1371/journal.pone.0124668 25861033 10.1371/journal.pone.0124668 PMC4393292

[4] Wang M, Hu RY, Wu HB, Pan J, Gong WW, Guo LH, et al (2015) Cancer risk among patients with type 2 diabetes mellitus: a population-based prospective study in China. Sci Rep 5: 11503 doi: 10.1038/srep11503 26082067 10.1038/srep11503 PMC4469976

[5] Ferraldeschi R, Welti J, Luo J, Attard G, de Bono JS (2015) Targeting the androgen receptor pathway in castration-resistant prostate cancer: progresses and prospects. Oncogene 34: 1745–1757. doi: 10.1038/onc.2014.115 24837363 10.1038/onc.2014.115 PMC4333106

[6] Fallowfield L, Payne H, Jenkins V (2016) Patient-reported outcomes in metastatic castration-resistant prostate cancer. Nat Rev Clin Oncol 13: 643–650. doi: 10.1038/nrclinonc.2016.100 27349193 10.1038/nrclinonc.2016.100

[7] Izumi K, Chang C (2013) Targeting inflammatory cytokines-androgen receptor (AR) signaling with ASC-J9® to better battle prostate cancer progression. Oncoimmunology 2: e26853 doi: 10.4161/onci.26853 24498558 10.4161/onci.26853 PMC3902114

[8] Thapa D, Ghosh R (2015) Chronic inflammatory mediators enhance prostate cancer development and progression. Biochem Pharmacol 94: 53–62. doi: 10.1016/j.bcp.2014.12.023 25593038 10.1016/j.bcp.2014.12.023

[9] Pin E, Stratton S, Belluco C, Liotta L, Nagle R, Hodge KA, et al (2016) A pilot study exploring the molecular architecture of the tumor microenvironment in human prostate cancer using laser capture microdissection and reverse phase protein microarray. Mol Oncol 10: 1585–1594. doi: 10.1016/j.molonc.2016.09.007 27825696 10.1016/j.molonc.2016.09.007 PMC5423125

[10] Xu HM, Wei W, Jia XY, Chang Y, Zhang L (2007) Effects and mechanisms of total glucosides of paeony on adjuvant arthritis in rats. J Ethnopharmacol 109: 442–448. doi: 10.1016/j.jep.2006.08.019 17000070 10.1016/j.jep.2006.08.019

[11] Chang Y, Wei W, Zhang L, Xu HM (2009) Effects and mechanisms of total glucosides of paeony on synoviocytes activities in rat collagen-induced arthritis. J Ethnopharmacol 121: 43–48. doi: 10.1016/j.jep.2008.09.028 18977427 10.1016/j.jep.2008.09.028

[12] Xu XX, Qi XM, Zhang W, Zhang CQ, Wu XX, Wu YG, et al (2014) Effects of total glucosides of paeony on immune regulatory toll-like receptors TLR2 and 4 in the kidney from diabetic rats. Phytomedicine 21: 815–823. doi: 10.1016/j.phymed.2013.12.003 24462407 10.1016/j.phymed.2013.12.003

[13] Zhu Q, Qi X, Wu Y, Wang K (2016) Clinical study of total glucosides of paeony for the treatment of diabetic kidney disease in patients with diabetes mellitus. Int Urol Nephrol 48: 1873–1880. doi: 10.1007/s11255-016-1345-5 27342654 10.1007/s11255-016-1345-5

[14] Zhou Z, Lin J, Huo R, Huang W, Zhang J, Wang L, et al (2012) Total glucosides of paeony attenuated functional maturation of dendritic cells via blocking TLR4/5 signaling in vivo. Int Immunopharmacol 14: 275–282. doi: 10.1016/j.intimp.2012.07.012 22846756 10.1016/j.intimp.2012.07.012

[15] Wang Y, Zhang H, Du G, Wang Y, Cao T, Luo Q, et al (2016) Total glucosides of paeony (TGP) inhibits the production of inflammatory cytokines in oral lichen planus by suppressing the NF-κB signaling pathway. Int Immunopharmacol 36: 67–72. doi: 10.1016/j.intimp.2016.04.010 27107800 10.1016/j.intimp.2016.04.010

[16] Zheng YQ, Wei W (2005) Total glucosides of paeony suppresses adjuvant arthritis in rats and intervenes cytokine-signaling between different types of synoviocytes. Int Immunopharmacol 5: 1560–1573. doi: 10.1016/j.intimp.2005.03.010 16023608 10.1016/j.intimp.2005.03.010

[17] Al-Asmari AK, Riyasdeen A, Abbasmanthiri R, Arshaduddin M, Al-Harthi FA. (2016) Scorpion (Androctonus bicolor) venom exhibits cytotoxicity and induces cell cycle arrest and apoptosis in breast and colorectal cancer cell lines. Indian J Pharmacol 48: 537–543. doi: 10.4103/0253-7613.190742 27721540 10.4103/0253-7613.190742 PMC5051248

[18] Khan A, Gillis K, Clor J, Tyagarajan K (2012) Simplified evaluation of apoptosis using the Muse cell analyzer. Postepy Biochem 58: 492–496. 23662443

[19] Ito Y, Ishiguro H, Kobayashi N, Hasumi H, Watanabe M, Yao M, et al (2015) Adipocyte-derived monocyte chemotactic protein-1 (MCP-1) promotes prostate cancer progression through the induction of MMP-2 activity. Prostate 75: 1009–1019. doi: 10.1002/pros.22972 25917126 10.1002/pros.22972

[20] Hu S, Delorme N, Liu Z, Liu T, Velasco-Gonzalez C, Garai J, et al (2010) Prosaposin down-modulation decreases metastatic prostate cancer cell adhesion, migration, and invasion. Mol Cancer 9: 30 doi: 10.1186/1476-4598-9-30 20132547 10.1186/1476-4598-9-30 PMC2825248

[21] Xia MZ, Liang YL, Wang H, Chen X, Huang YY, Zhang ZH, et al (2012) Melatonin modulates TLR4-mediated inflammatory genes through MyD88- and TRIF-dependent signaling pathways in lipopolysaccharide-stimulated RAW264.7 cells. J Pineal Res 53:325–334. doi: 10.1111/j.1600-079X.2012.01002.x 22537289 10.1111/j.1600-079X.2012.01002.x

[22] Nabbi A, Riabowol K (2015) Rapid Isolation of Nuclei from Cells In Vitro. Cold Spring Harb Protoc 2015: 769–772. doi: 10.1101/pdb.prot083733 26240403 10.1101/pdb.prot083733

[23] Suzuki K, Bose P, Leong-Quong RY, Fujita DJ, Riabowol K (2010) REAP: A two minute cell fractionation method. BMC Res Notes 3: 294 doi: 10.1186/1756-0500-3-294 21067583 10.1186/1756-0500-3-294 PMC2993727

[24] Smith PC, Hobisch A, Lin DL, Culig Z, Keller ET (2001) Keller, Interleukin-6 and prostate cancer progression. Cytokine Growth Factor Rev 12: 33–40. 11312117 10.1016/s1359-6101(00)00021-6

[25] Mistry T, Digby JE, Desai KM, Randeva HS (2007) Obesity and prostate cancer: a role for adipokines. Eur Urol 52: 46–53. doi: 10.1016/j.eururo.2007.03.054 17399889 10.1016/j.eururo.2007.03.054

[26] Guo Y, Xu F, Lu T, Duan Z, Zhang Z (2012) Interleukin-6 signaling pathway in targeted therapy for cancer. Cancer Treat Rev 38: 904–910. doi: 10.1016/j.ctrv.2012.04.007 22651903 10.1016/j.ctrv.2012.04.007

[27] Michalaki V, Syrigos K, Charles P, Waxman J (2004) Serum levels of IL-6 and TNF-alpha correlate with clinicopathological features and patient survival in patients with prostate cancer. Br J Cancer 90: 2312–2316. doi: 10.1038/sj.bjc.6601814 15150588 10.1038/sj.bjc.6601814 PMC2409519

[28] Stark JR, Li H, Kraft P, Kurth T, Giovannucci EL, Stampfer MJ, et al (2009) Circulating prediagnostic interleukin-6 and C-reactive protein and prostate cancer incidence and mortality. Int J Cancer 124: 2683–2689. doi: 10.1002/ijc.24241 19189403 10.1002/ijc.24241 PMC2667697

[29] Lin DL, Whitney MC, Yao Z, Keller ET (2001) Interleukin-6 induces androgen responsiveness in prostate cancer cells through up-regulation of androgen receptor expression. Clin Cancer Res 7: 1773–1781. 11410519

[30] Lee SO, Lou W, Hou M, de Miguel F, Gerber L, Gao AC (2003) Interleukin-6 promotes androgen-independent growth in LNCaP human prostate cancer cells. Clin Cancer Res 9: 370–376. 12538490

[31] Wallner L, Dai J, Escara-Wilke J, Zhang J, Yao Z, Lu Y, et al (2006) Inhibition of interleukin-6 with CNTO328, an anti-interleukin-6 monoclonal antibody, inhibits conversion of androgen-dependent prostate cancer to an androgen-independent phenotype in orchiectomized mice. Cancer Res 66: 3087–3095. doi: 10.1158/0008-5472.CAN-05-3447 16540658 10.1158/0008-5472.CAN-05-3447

[32] Murphy C, McGurk M, Pettigrew J, Santinelli A, Mazzucchelli R, Johnston PG, et al (2005) Nonapical and cytoplasmic expression of interleukin-8, CXCR1, and CXCR2 correlates with cell proliferation and microvessel density in prostate cancer. Clin Cancer Res 11: 4117–4127. doi: 10.1158/1078-0432.CCR-04-1518 15930347 10.1158/1078-0432.CCR-04-1518

[33] Caruso DJ, Carmack AJ, Lokeshwar VB, Duncan RC, Soloway MS, Lokeshwar BL (2008) Osteopontin and interleukin-8 expression is independently associated with prostate cancer recurrence. Clin Cancer Res 14: 4111–4118. doi: 10.1158/1078-0432.CCR-08-0738 18593988 10.1158/1078-0432.CCR-08-0738 PMC2665724

[34] Sharma J, Gray KP, Harshman LC, Evan C, Nakabayashi M, Fichorova R, et al (2014) Elevated IL-8, TNF-α, and MCP-1 in men with metastatic prostate cancer starting androgen-deprivation therapy (ADT) are associated with shorter time to castration-resistance and overall survival. Prostate 74: 820–828. doi: 10.1002/pros.22788 24668612 10.1002/pros.22788

[35] Inoue K, Slaton JW, Eve BY, Kim SJ, Perrotte P, Balbay MD, et al (2000) Interleukin 8 expression regulates tumorigenicity and metastases in androgen-independent prostate cancer. Clin Cancer Res 6: 2104–2119. 10815938

[36] Araki S, Omori Y, Lyn D, Singh RK, Meinbach DM, Sandman Y, et al (2007) Interleukin-8 is a molecular determinant of androgen independence and progression in prostate cancer. Cancer Res 67: 6854–6862. doi: 10.1158/0008-5472.CAN-07-1162 17638896 10.1158/0008-5472.CAN-07-1162

[37] Maxwell PJ, Coulter J, Walker SM, McKechnie M, Neisen J, McCabe N, et al (2013) Potentiation of inflammatory CXCL8 signalling sustains cell survival in PTEN-deficient prostate carcinoma. Eur Urol 64: 177–188. doi: 10.1016/j.eururo.2012.08.032 22939387 10.1016/j.eururo.2012.08.032 PMC4185293

[38] Maxwell PJ, Neisen J, Messenger J, Waugh DJ (2014) Tumor-derived CXCL8 signaling augments stroma-derived CCL2-promoted proliferation and CXCL12-mediated invasion of PTEN-deficient prostate cancer cells. Oncotarget 5: 4895–4908. doi: 10.18632/oncotarget.2052 24970800 10.18632/oncotarget.2052 PMC4148108

[39] Karin M (2009) NF-kappaB as a critical link between inflammation and cancer. Cold Spring Harb Perspect Biol 1: a000141 doi: 10.1101/cshperspect.a000141 20066113 10.1101/cshperspect.a000141 PMC2773649

[40] McCall P, Bennett L, Ahmad I, Mackenzie LM, Forbes IW, Leung HY, et al (2012) NFκB signalling is upregulated in a subset of castrate-resistant prostate cancer patients and correlates with disease progression. Br J Cancer 107: 1554–1563. doi: 10.1038/bjc.2012.372 23093296 10.1038/bjc.2012.372 PMC3493754

[41] Jin RJ, Lho Y, Connelly L, Wang Y, Yu X, Saint Jean L, et al (2008) The nuclear factor-kappaB pathway controls the progression of prostate cancer to androgen-independent growth. Cancer Res 68: 6762–6769. doi: 10.1158/0008-5472.CAN-08-0107 18701501 10.1158/0008-5472.CAN-08-0107 PMC2840631

[42] Jin R, Yi Y, Yull FE, Black well TS, Clark PE, Koyama T, et al (2014) NF-κB gene signature predicts prostate cancer progression. Cancer Res 74: 2763–2772. doi: 10.1158/0008-5472.CAN-13-2543 24686169 10.1158/0008-5472.CAN-13-2543 PMC4024337

[43] Zhang Y, Huang H, Zhou H, Du T, Zeng L, Cao Y, et al (2014) Activation of nuclear factor κB pathway and downstream targets survivin and livin by SHARPIN contributes to the progression and metastasis of prostate cancer. Cancer 120: 3208–3218. doi: 10.1002/cncr.28796 24925528 10.1002/cncr.28796

[44] Huang S, Pettaway CA, Uehara H, Bucana CD, Fidler IJ (2001) Blockade of NF-kappaB activity in human prostate cancer cells is associated with suppression of angiogenesis, invasion, and metastasis. Oncogene 20: 4188–4197. doi: 10.1038/sj.onc.1204535 11464285 10.1038/sj.onc.1204535

[45] Burger M, Hartmann T, Burger JA, Schraufstatter I. (2005) KSHV-GPCR and CXCR2 transforming capacity and angiogenic responses are mediated through a JAK2-STAT3-dependent pathway. Oncogene 24:2067–75. doi: 10.1038/sj.onc.1208442 15688008 10.1038/sj.onc.1208442

[46] Jayatilaka H, Tyle P, Chen JJ, Kwak M, Ju J, Kim HJ, et al (2017) Synergistic IL-6 and IL-8 paracrine signalling pathway infers a strategy to inhibit tumour cell migration. Nat Commun 8: 15584 doi: 10.1038/ncomms15584 28548090 10.1038/ncomms15584 PMC5458548

[47] Barton BE, Karras JG, Murphy TF, Barton A, Huang HF (2004) Signal transducer and activator of transcription 3 (STAT3) activation in prostate cancer: Direct STAT3 inhibition induces apoptosis in prostate cancer lines. Mol Cancer Ther 3: 11–20. 14749471

[48] Huang HF, Murphy TF, Shu P, Barton AB, Barton BE (2005) Stable expression of constitutively-activated STAT3 in benign prostatic epithelial cells changes their phenotype to that resembling malignant cells. Mol Cancer 4: 2 doi: 10.1186/1476-4598-4-2 15647107 10.1186/1476-4598-4-2 PMC546221

[49] Gu L, Talati P, Vogiatzi P, Romero-Weaver AL, Abdulghani J, Liao Z, et al (2014) Pharmacologic suppression of JAK1/2 by JAK1/2 inhibitor AZD1480 potently inhibits IL-6-induced experimental prostate cancer metastases formation. Mol Cancer Ther 13: 1246–1258. doi: 10.1158/1535-7163.MCT-13-0605 24577942 10.1158/1535-7163.MCT-13-0605 PMC4013199

[50] Ni Z, Lou W, Leman ES, Gao AC (2000) Inhibition of constitutively activated Stat3 signaling pathway suppresses growth of prostate cancer cells. Cancer Res 60: 1225–1228. 10728680

[51] Gao L, Zhang L, Hu J, Li F, Shao Y, Zhao D, et al(2005) Down-regulation of signal transducer and activator of transcription 3 expression using vector-based small interfering RNAs suppresses growth of human prostate tumor in vivo. Clin Cancer Res 11: 6333–6341. doi: 10.1158/1078-0432.CCR-05-0148 16144938 10.1158/1078-0432.CCR-05-0148

[52] Zhang L, Gao L, Li Y, Lin G, Shao Y, Ji K, et al (2008) Effects of plasmid-based Stat3-specific short hairpin RNA and GRIM-19 on PC-3M tumor cell growth. Clin Cancer Res 14: 559–568. doi: 10.1158/1078-0432.CCR-07-1176 18223232 10.1158/1078-0432.CCR-07-1176

[53] Canesin G, Evans-Axelsson S, Hellsten R, Sterner O, Krzyzanowska A, Andersson T, et al (2016) The STAT3 Inhibitor Galiellalactone Effectively Reduces Tumor Growth and Metastatic Spread in an Orthotopic Xenograft Mouse Model of Prostate Cancer. Eur Urol 69: 400–404. doi: 10.1016/j.eururo.2015.06.016 26144873 10.1016/j.eururo.2015.06.016

[54] Zhang J, Ahn KS, Kim C, Shanmugam MK, Siveen KS, Arfuso F, et al (2016) Nimbolide-Induced Oxidative Stress Abrogates STAT3 Signaling Cascade and Inhibits Tumor Growth in Transgenic Adenocarcinoma of Mouse Prostate Model. Antioxid Redox Signal 24: 575–589. doi: 10.1089/ars.2015.6418 26649526 10.1089/ars.2015.6418

